# Case Study Quasi-Qualitative Analysis of Peer Group Supervision of a Child Trauma Recovery Program in Occupied Palestine

**DOI:** 10.1007/s40653-016-0127-7

**Published:** 2017-02-01

**Authors:** Ian G. Barron, Ghassan Abdallah, Unni Heltne

**Affiliations:** 1 School of Education and Social Work, University of Dundee, Nethergate Dundee, UK; 2 Center for Applied Research in Education, Ramallah, Israel; 3 Centre for Crisis Psychology, Bergen, Norway

**Keywords:** Adolescents, Traumatization, Group supervision, Counseling, Evaluation

## Abstract

This case study explores the impact of peer group supervision (PGS) for counselors delivering Teaching Recovery Techniques (TRT), a group psycho-social program for traumatized adolescents. Interviews were conducted with seven school counselors, from villages near East Jerusalem in occupied Palestine; a skilled supervisor; and an international trainer. A quasi-qualitative analysis was conducted to quantify responses and themes along with a measure of inter-rater reliability. Participants reported that even in a context of military violence, PGS provided a reflective process inclusive of formative, normative and restorative functions. Locally available PGS was viewed as essential within a geographically fragmented context. Counselors’ reported PGS led to a trusting environment in contrast to military practices and gender norms. War stressors and lack of Training of Trainers led to low counselor confidence in training others in TRT. PGS and Trainer of Trainers were recommended as core aspects of program delivery and to support training of colleagues.

Peer group supervision (PGS) has been used with a range of professions, including occupational therapy, nursing, social work and psychology and professional levels, such as managers, supervisors and practitioners (Spence et al. 2001). Despite PGS being widely used in practice, research has remained limited (Mastoras and Andrews 2011). Evaluative studies tend to focus on students studying for professional practice (Bjorke 2013), post-qualifying professionals (Remley et al. 1987) and private practitioners (Lewis et al. 1988). Most studies report a range of positive effects (Lakeman and Glasgow 2009).

PGS has been defined as “the regular meeting of a group of supervisees (a) with a designated supervisor or supervisors, (b) to monitor the quality of their work, and (c) to further their understanding of themselves as clinicians, of the clients with whom they work, and of service delivery in general. These supervisees are aided in achieving these goals by their supervisor(s) and by their feedback from and interactions with each other” (Bernard and Goodyear 2009, p.244). Within occupied Palestine, the compartmentalized nature of the West Bank with restrictions on movement, and the need to apply for travel permits (Barron et al. 2013), has limited access to any skilled supervision.

The aims of PGS include the development of professional knowledge/skills, values, identity and the development of a network to reduce counselor isolation and burn out (Certo 2012). Counselors report a wide range of benefits including: sharing experience; reflection and problem-solving; new perspectives; skills practice; emotional support; and the development of cooperation and communication. Benefits also accrue to organizations, with the growth of cultures of appreciation, enhanced knowledge/skill and flexibility (McMahon and Patton 2000). Corey (2011) suggests PGS is a cost-effective way for organizations to support staff. Counselors have identified a number of factors for successful delivery, including: the centrality of a reflective process (Corey 2011); supervisee sense of safety (Fleming et al. 2010); confidentiality and trust; clarity of time and place; mutual support; the positive resolution of conflict and shared learning (Rutter 2006). Page and Wosket (2001) suggest the size and structure of groups are important to facilitate self-disclosure and feedback. Closed groups of three to six participants are recommended.

A range of supervision models for PGS have been suggested. Baruch (2009) identified seven; however, most therapeutic paradigms have developed a model of supervision based on the theory and methods of therapy, such as solution focused, cognitive-behavioral, psychodynamic, developmental group process and counselor/client role centered supervision (Lassiter et al. 2008). The congruity of therapeutic and supervision model helps the transfer of experiential learning to practice (Bradley and Ladany 2001). The current study therefore, utilized the same CBT model for program delivery and PGS. PGS compared to program delivery, however, is a context of greater intimacy and trust, where counselors can be challenged to disclose their thoughts and feelings more so than adolescents in a group (Bradley and Ladany 2001).

Hawkins and Shohet (2006) describe the three functions of supervision as formative, normative and restorative. The formative function involves educating counselors about the client group, their context and the nature of program delivery. In Palestine, this includes the needs of adolescents and families living under occupation; the impact of trauma on adolescents and counselors; and the challenges of delivering a program within a context of military violence. Program factors include understanding theoretical foundations; implementing protocols; program adaptation and evaluation (Joubert et al. 2013). The normative function of supervision aims to support counselors to adhere to professional, program and evaluation protocols with some supervision groups being accredited for continuing professional learning (Thomasgard and Collins 2003). Ethical practice is often enshrined in codes of professional behavior including respect, genuineness, trust, choice, empathic engagement and self-responsibility (Corey et al. 2014). Many professions require evidence of supervision attended over a stipulated number of hours. Codes of practice, however, are not without their problems. The prescribing of professional behavior can lead to overly bureaucratic practice, which can undermine counselor/client relationship (Fenton 2014). PGS, however, can provide a context to help counselors reflect and resolve such incongruities.

The restorative function of PGS involves creating an emotional and physically safe environment to facilitate reflection (Shulman 2010). Counselors can be helped to notice signs of stress, trauma or burn out within themselves. Anxiety, helplessness, depersonalisation, de-realisation, somatic symptoms and flashbacks are all signs of concern (Cohen and Collens 2013). Counselors can be supported to identify strategies and if necessary, support services. Delivering trauma-specific programs can trigger personal issues that need to be addressed, not in supervision, but in therapy (Trippany et al. 2004). Counselor self-care is also part of the restorative function. Time with friends and family, hobbies, and setting boundaries on work/life balance are all important (Skovholt and Trotter-Mathison 2014).

PGS, however, is not without its problems. Counselman and Weber (2004) identified supervisee shame and guilt as barriers to self-disclosure. These occur as a consequence of the lack of challenge for the counselor to take risks in sharing thoughts and feelings. Poor relational contracting can also undermine the effectiveness of PGS. Lack of leadership, irregular attendance, frequent lateness, and inappropriate turn-taking can all be undermining. Proctor (2004) highlighted that coercive communication can occur among peers. As a consequence, individual supervision, in addition to PGS, may be advisable (Akhurst and Kelly 2006).

## The Current Study

No study to date has explored the effect of PGS for counselors delivering a trauma recovery program in a situation of violent military occupation. The current case study sought to address this omission by introducing and evaluating PGS for school counselors delivering a CBT group-based trauma recovery program (Teaching Recovery Techniques: TRT) to adolescents in occupied Palestine. Counselors were experiencing isolation and low levels of confidence in a compartmentalized geographical context. In addition to the three functions of PGS, it was hoped supervision might encourage counselors to have the confidence to train other West Bank counselors in TRT, an explicit aim of TRT training. Hitherto they had been reluctant to do so. As outcome evaluations delivered too soon can undermine promising initiatives (Barron and Topping 2011), an exploratory qualitative case study approach was utilized. Semi-structured interviews were held with stakeholders in the project (counselors, skilled supervisor and international trainer) to assess counselor experience of PGS.

## Methods

### Study Design

The case study had two goals: 1) to explore counselor, supervisor and trainer perception of PGS in supporting TRT delivery to adolescents in the violent military context of occupied Palestine, and 2) to seek counselor perception of whether PGS increased the likelihood of them delivering TRT training to other counselors. As noted previously, the latter is an assumption within TRT training. As PGS was a new initiative, a qualitative approach using semi-structured interviews was taken to assessing participant experience, perception of impact and identification of future supervision needs. The study sought to compare and contrast the multiplicity of perspectives from participants and explore the singularity and uniqueness of participant experience. Research ethics approval was through a UK University Research Ethics Committee (UREC). This required active informed consent from the Palestinian Authority Ministry of Education, counselors, skilled supervisor and the Children and War Foundation TRT trainer. The latter is a non-governmental organization (NGO) established to develop empirically-based trauma recovery programs for children in disaster and war contexts.

### Participants

The recruitment strategy involved purposive selection of (i) school counselors (*n* = 10), from a corpus sample of 18, who had experienced PGS to facilitate TRT delivery (Smith et al. 2008); (ii) the skilled supervisor, a Palestinian educational psychologist who held an overview of counselor practice and (iii) the Children and War Foundation trainer, who held a view of training across countries. Ten school counselors from five schools in five different villages along the separation wall near East Jerusalem were invited to participate. Counselors were selected because of the high levels of military violence experienced in these school areas (Barron et al. 2016). Counselors experienced PGS with others counselors in the same areas (other participants). Counselors were from two professions, social work (*n* = 5) and psychology (*n* = 5), with five males and five females. Counselor skill levels were reported, by the skilled supervisor, as based on theoretical knowledge from 1 year’s University training rather than skilled practice. Counselors were initially approached by the field researcher via telephone and invited to participate in the study. The trainer and supervisor were approached by email by the principal researcher.

### Researchers

In qualitative research it is important to make explicit the position and potential bias of researchers (Orne and Bell 2015). Two researchers were involved in the design and delivery of the study and a research assistant was subsequently involved in inter-rater reliability analysis. This provided another independent view of the data. The two researchers included a reader in trauma studies at a UK University and a director of a NGO dedicated to peace building in occupied Palestine. The researchers are experienced and accredited educational psychologists, one in the UK, the other in Palestine. The research assistant was a theology of religion graduate working with a refugee NGO in Scotland and who had previously worked in the West Bank for 18 months. The two researchers are experienced in training and delivering supervised professional practice. PGS training was delivered by the researchers. The Palestinian researcher, was the skilled supervisor in the study. Issues of bias are addressed in the discussion.

### Program

Posttraumatic stress is a pervasive problem in Palestine (Barron et al. 2013). TRT, developed by the Children and War Foundation, doubles as a training program for delivering TRT to adolescents and as a Trainer of Trainers. TRT has a developing evidence base for reducing posttraumatic stress in 11-18 years olds in disaster situations and contexts of war (Barron et al. 2013, 2016; Ehntholt et al. 2005). The program, delivered to adolescents in groups of ten, is based on cognitive-behavioral theory, and is delivered over five sessions. The initial session enables adolescents to understand that the trauma reaction is normal in extreme circumstances and teaches adolescents how to feel emotionally safe. Subsequent sessions teach adolescents a range of coping skills to stop intrusive images, sounds or smells; reduce hyper-arousal through stabilization and relaxation techniques; and desensitize phobic avoidance behavior through use of relaxation with anxiety and anger hierarchies. A three-day training for the eighteen counselors included information on: normalizing the trauma reaction, working through case studies and experiencing the same activities as adolescents to learn coping skills for flashbacks, hyperarousal and avoidance.

### Supervision

To support high standards of program delivery and encourage counselors to train other counselors in TRT, skills’ training was provided in PGS (Proctor 2004). Counselors were expected to meet monthly with other counselors in the same area for an hour in pairs/small groups during the 6 month project to: prepare and reflect on program delivery; cope emotionally; follow program protocols; make program adaptations within theoretical guidelines; and assess their continuing professional development needs. Supervision sessions held in local schools negated the need for counselors to apply for travel permits, frequently denied by Israeli Authorities (OCHA 2013). The model of supervision involved peers taking turns at leading sessions. Two PGS sessions, however, were led by a skilled supervisor and occurred during the second and fourth months of the project. These session lasted 90 min each and followed the same process as other PGS sessions. A higher level of advice was provided by the skilled supervisor. A one-day PGS training was delivered by the two researchers. Training emphasized the importance of attendance, timings, closed membership, record keeping, clarity of leadership, rules, turn-taking, respect, self-disclosure, reciprocal support and permission to challenge. PGS training was based on TRT group process and involved creating an active, positive group environment; normalizing and labelling reactions; the promotion of self-efficacy and coping skills; and modeling and role play of TRT activities with feedback. The tasks of supervision were educative (what do I need to know about trauma and the TRT program), restorative (reflecting on feelings) and normative (holding to program protocols and adaptations within theoretical guidelines).

### Interviews

Interviews followed a semi-structured format. The field researcher conducted the interviews with counselors a month following the end of the project. Pre-supervision interviews were not held because of budgetary constraints. The principal researcher conducted the interviews with the skilled supervisor and trainer. Interviews lasted 45 min on average and were held at the end of the school day. All interviews were conducted by telephone and digitally recorded. Questions, based on issues and gaps identified in the literature, included: aims of supervision; spontaneously reported gains; changes in understanding; protocol adherence; impact on ethical practice; effect on stress; challenges; the negative aspects of supervision; the nature of future TRT delivery; the likelihood of TRT training to other counselors; and recommendations for future supervision. Counselors were requested to rate gains from 0 to 10, where 0 was no gain and 10 was a substantial gain.

### Analysis

All data was transcribed in Arabic and translated into English by a Palestinian interpreter. A quasi-qualitative approach was taken to analyzing the data (Braun and Clarke 2006). This six-step approach involved familiarization with the whole data set; initial identification of codes of meaning from statements; reviewing and adapting codes and related statements and the initial identification of themes; counting the number of statements under each code; revisiting statements, codes and themes for accuracy; and drafting the report that afforded another review of codes, statements within codes and themes. The approach was iterative, where researcher and research assistant sought to discover meanings from participants own words. Codes and themes were identified for similarity, difference and doubt within and between counselors, supervisor and trainer. An aim was to make data accessible for contestability by providing codes and themes in participants’ own words, as well as providing small and large quotes to exemplify themes. In the discussion section, the analysis of data is compared with pre-existing categories about supervision from the research. Inter-rater reliability involved the principal investigator and research assistant independently analyzing participant statements, codes and themes. This inter-rater reliability is reported as Cohen’s Kappa *k*. for collated themes.

## Results

### Extent of Participation

Two social workers (males) and one psychologist (female) did not attend for interview. Reasons were increased settler violence, excessive work demands, school closure and non-payment of salary. Counselors attended 5.4 peer PGS sessions (4 to 7 range) on average, during a 6 month period. Sessions occurred before, during and after program delivery. The average length of session was 46 min, ranging from 40 to 60 min. All counselors’ experienced face to face supervision and three, in addition, experienced phone supervision. As one counselor commented “PGS was possible with counselors close by.” Five counselors experienced PGS led by the skilled supervisor. Sessions lasted 80 min and were held in Ministry of Education offices. Two counselors attended two sessions; five counselors attended one session. Table 1 shows the comparative benefits of PGS identified by participants. Patterns of commonality and distinctive contributions by counselors, supervisor and trainer are evident in the codes and themes. Across all questions inter-rater reliability was high and at a satisfactory level for qualitative studies (*k* = .94). The research assistant identified ‘receiving and giving feedback’ as part of ‘skill gain’ benefit and framed the ‘knowledge and skill’ barrier theme as competency issues. There was some uncertainty whether some comments were knowledge or skill gains) e.g. making appropriate responses).

**Table 1:** PGS themes: perceived benefits

Themes^a^	Codes	Exemplar Statements	By whom
Participation	*m* = 5.4 sessions	“PGS was possible with colleagues close by”	C
PGS aims and functions	clarification; seeking assurance	“clarifying program procedures and content”	C, S, T
	problem solving; reflection; emotional reaction﻿s	“time for reflection”; “emotional reaction”	C, T
Space to share and practice	practice; learn from others;	“practicing with colleagues”	C, S, T
	exchange ideas; ask questions	“opportunity to learn from others”	C, S
Gains in understanding	appropriate response; better informed; clear expectations	“Knowing the way to respond to need/questions”	C, S
Skill gains	listening; engagement; giving time to talk; safe and relaxed group; TRT easier to do	“Better at making the group feel relaxed”	C, S
		.	
Developing ethical practice	Listening to children; caring; respectful; professional limits	“Not doing things professionally not skilled to do”	C, T
Improved protocol adherence	7.29 (C) vs. 5 (S) out of 10	“A new hope they will?”	C, S, T
Increased confidence	7.28 (C) vs 7.5 (S)	“They felt they were doing something important”	C, S
Reduced stress	7.1 (C) vs. 8 (S)	“It was in their faces … ways of talking”	C, S, T

*C* counsellor, *S* supervisor, *T* trainer

^a^Additional supervisor themes – evaluative practice; culture of appreciation; communication across geographical areas; relationships between organisations; and PGS challenged cultural norms

### PGS Aims and Functions

Counselors reported five PGS aims: “clarifying program content and procedures” (*n* = 4 statements); “checking doing the right thing” (*n* = 4); problem-solving (*n* = 3); “sharing work done” (*n* = 2); and “time for reflection” (*n* = 1). The supervisor identified similar aims: “follow up on how things were going; finding out what counselors are facing and the need to overcome these” (*n* = 2). This included “questions about implementation and not enough time with students in school” which resulted in “lessons delivered after school.” The supervisor, however, took a longer term view of progress than counselors, “I think the noticeable difference may be by the second semester” (*n* = 2). The trainer, as with the counselors and supervisor, held the aim of PGS as formative, “local supervisors was a great advantage, because supervision could clarify things.” The trainer also recognized the inter-relationship between formative and restorative functions, “Counselors were sharing the same information from training and getting a sense of being in it together … some participants had an emotional reaction at training and approached me.” In response they received “more clarification and feedback on techniques to address stress … and participants were encouraged to form a group … the importance of local coordination for this was emphasized.” For counselors new to delivering TRT and PGS, frequency of comments suggests aims were mostly formative (learning from each other and a skilled supervisor) and normative (am I doing it well enough) and to a lesser extent, restorative (a sense of being in it together).

### PGS Provided Opportunity to Share and Practice

Counselors valued “practicing with colleagues” (*n* = 5); “the opportunity to learn from others (*n* = 3); “meeting colleagues to exchange ideas” (*n* = 3); “share what done” (*n* = 2); “time for reflection” (*n* = 2) and “asking about small things.” The supervisor, however, identified the need for PGS as ongoing, “Many questions were raised and discussed and the number of inquiries showed they still need more opportunity for this”.

### Gains in Understanding/Skills

All counselors reported that PGS led to increases in understanding (8.42 average; six to eight range) and “improved communication skills” with adolescents (*n* = 10). The supervisor rated the group “around 8, at least they now reflect, listen to each other, listen to somebody else.” Counselors reported gains in understanding as: “knowing the right ways to respond to group needs and questions” (*n* = 10); being “better informed” (*n* = 7); and clearer about what was expected. Growth in skills included: “active listening” (*n* = 5), “respecting and engaging all” (*n* = 4); “giving students more time to talk and understand” (*n* = 2); “better at making the group feel relaxed” (*n* = 2); and “more aware students feeling the group was under control” (*n* = 1). One counselor reported TRT was “easier to do as a result.” Knowledge and skill gains reflected a balance of formative and normative functions.

The trainer (T) and supervisor (S) reiterated the value of PGS for clarifying practice. “Local peer supervision was a great advantage because supervision could clarify what to do (T)” and “participants gained reassurance … sharing and hearing each other’s experiences and experiencing that they were in it together” (*n* = 3). The supervisor also reported counselors “felt the seriousness of the work. Not like what others are doing, they experienced serious follow-up.”

### Gains in Ethical Practice

Counselors reported gains in ethical practice at 7.75 on average (seven to eight range). The supervisor rated the group 7. Counselors emphasized their learning of how important it is to “listen to children (*n* = 5).” The trainer reported training placed emphasis on “respectful caring and not doing things not professionally skilled to do, recognizing their own limits.” The supervisors comments were different again and referred to the counselors’ process of engagement, consistency and predictability and keeping the group process safe for all as, “Counselors were very committed to this (ethical practice): talking about informing parents; respecting parents and students; invitations in good time; not changing meeting time; making sure no student interrupting others, using insulting words, and not contradicting each other.” It appears, counselors experienced gains in ethical practice that bridged both formative and normative functions.

### Improved Protocol Adherence

The normative function of protocol adherence was “emphasized in training” (T). Counselors rated protocol adherence following PGS as 7.29, ranging from six to eight. Counselors rated adapting protocols within theoretical guidelines similarly at 7.12, ranging from seven to eight. The supervisor, rating 5, was less confident of counselor capacity, expressing, “Not so much, a new hope that they will.” It is, therefore, uncertain the extent to which this normative function of PGS was achieved. A more objective measure of program fidelity before and after PGS would be helpful to assess such a skill change.

## Increased Confidence and Reduced Stress

In terms of PGS providing a restorative function, counselors reported an increase in confidence and a reduction in stress. All counselors rated increases in confidence (average 7.28; six to eight range), for example, “It made it easier, I felt more confident about delivering the program.” The supervisor rated counselors, who attended skilled supervision, similarly at 7.5 on average but noted “they were not all at the same level of confidence. Some counselors were at least a 9, because of their different experiences, social cultural background, and different levels of commitment … PGS helped raise counselor self-esteem and confidence. I think they felt they are doing something important.” Counselors and supervisor perceived gains in counselor self-confidence was a consequence of PGS; however, there was a range of experience and this may have been related to significance of purpose, as well as gains in understanding and skills.

All counselors reported a significant reduction in stress, with an average rating of 7.1 with a narrow range from six to eight, reporting “I wasn’t so anxious with the students” and “I was more relaxed about delivering TRT.” The supervisor reported counselors “returned for a second session, they were not embarrassed, not feeling weak and it was in their faces, hand gestures and ways of talking.” He rated counselors as making higher gains in reduced stress (an eight) than counselors themselves. Reduced stress and increased confidence may or may not be related, but either way, PGS provided a restorative function that led to emotional gains for counselors.

### Additional Supervisor Themes

A further five themes were identified from the skilled supervisor. PGS was reported as encouraging: (i) evaluative practice (*n* = 4), as “Unfortunately evaluation is not something done much in my community, particularly in this field. We have managed to break through the mentality that it is not enough to be trained and implement, but also what is needed is evaluation”; (ii) a culture of appreciation (*n* = 1), given “Another thing is the high appreciation of the people involved in this training: trainer, researchers, supervisor and the Department of Counselling and the Minister of Education;” (iii) communication across geographical areas (*n* = 1), as it was noted that “Counselors contacted colleagues after the work was done in other areas. They are talking more across areas”; (iv) relationships between organizations (*n* = 6), as “PGS helped us discover the commitment … among counselors and the criteria for this, coming on time although it was after school day; asking lots of questions; not wasting time and keeping in touch. I think it helped a lot in building of trust between organizations” and (v) PGS challenged cultural norms (*n* = 3). For instance, “for a female to be out in the community, and to be ready to give her phone number is not an easy thing. To get a man’s phone number is easy. You need to realize how conservative the counselors are.” Finally, more in relation to TRT than supervision, the supervisor commented, “What I liked was none of them expressed that this program is against his/her social cultural background or even religious background,” possibly indicating how culturally congruent TRT was. Table 2 summarizes and compares the issues, themes and codes related to barriers of implementation for TRT and PGS reported by counselors, supervisor and trainer. Three core issues, six themes and twenty codes were identified. There was, however, less commonality of barrier codes and themes across counselors, supervisor and trainer than was evident in the benefits of PGS.

**Table 2:** Perceived barriers

Issue	Themes	Codes	By whom
Research non-participation	context of occupation	settler violence; school closure; salary non-payment	C
Program delivery barriers	competency	self-doubt; time; confidence; confusion; superficial practice	C
		anxiety; limited theory; significant change in practice	S
	situational context	parental suspicion; military and settler violence; school closures	C, S
Barriers to training others	lack of knowledge & skill	insufficient knowledge; limited follow-up, encouragement and feedback	C, T
	lack of others’ interest	paradigm rigidity; counselors feeling excluded	S
		thinking trained counselor showing off	S
	lack of specific training	trainer of trainers’ recommendation	S

*C* counselor, *S* supervisor, *T* trainer

### Barriers to Program Delivery - Competency

Counselors reported: “new things raised doubt” (*n* = 1); “limited time” (*n* = 1); “being shy” (*n* = 1); “feeling unable to deliver” (*n* = 1); “By knowing about other work, I sometimes get confused” (*n* = 1); and “I keep watching and just write every small word” (*n* = 1). One counselor noted, “Practice felt superficial.” The supervisor reported “Counselors were so thirsty and hungry for responses to their needs. There were issues in understanding and acquiring how to implement TRT methodology.” Several counselors were scared to implement because they were “not confident until they knew it perfectly.” “We don’t want to be a curse instead of a blessing … she was afraid because of the high complexity of problems for students and because she was not very skillful, that she might do something wrong or deepen the trauma. Highly cautious is what I observed.” The supervisor reported PGS was “not so much about theory but in response to their questions and enquiries. Most of the time they pretend to understand the theory, however, the main challenges are the implementation, how to deliver. What they are used to is sitting with the student and filing in a form. In contrast counselors had to learn to work face to face, this was new for them. This was an unexpected challenge for them, the need to give extra effort and adaptation.” Counselors asked for more “practice amongst themselves.” The supervisor commented, “The focus of PGS was on being fully prepared, aware of the students’ situation, aware of the issues in your area and do what you can do to increase your confidence and self-esteem. If they are very religious, they pray before they go into the session.”

### Barriers to Program Delivery - Situational Context

Some parents were reported as barriers to TRT, as they were “suspicious of why the program was being run”; others were “hesitant about involvement, as they did not want children to be late after school because of settler violations. For example, one female student, 12 years old, was stopped by a female Israeli soldier and delayed her from going home after she beat her up. This girl was in the media.” And finally the supervisor reported “all the counselors were victims of unpaid salaries, payments and strikes.” This apparently led to “fewer opportunities to practice skills.” Barriers to program delivery, therefore, included parental uncertainty, sessions after school increasing risks for adolescents; and school closures leading to less opportunities to practice.

### Barriers to Training Others

Counselors rated 5.6 on average (4–6 range), for likely to train others. Comments included: “I need more follow up” (*n* = 3) and “I want to hear and know more” (*n* = 2). A range of barriers were identified by the supervisor, as it was noted that “some are trained in other programs and don’t want to leave other sources of knowledge. Other counselors heard about the training and questioned why they were not included. Some counselors were not able to convey TRT knowledge and skills to them. Culturally, the offer of training was misconstrued at a personal level, it was seen as showing off.” Positively, the supervisor concluded, “some believe in team work and have professional awareness. Seriousness and strict follow-up by Department of Counseling makes me feel more optimistic.”

According to the trainer, the challenge of counselors training other counselors has been a “major issue.” Counselors were seen as “over estimating … feeling they have to be very skilled to train others,” whereas “these are people experienced with children, dealing with children all the time, when they start to train adults they feel they have to be brilliant.” In addition, the trainer reflected on the “negative impact of the separation wall and checkpoints involved in travelling around the West Bank. As a consequence this causes very real practical problems in delivering training in different locations.” In summary, barriers to delivering training included: counselors perceived lack of knowledge and skill, need for more support; other counselors not wanting trained for various reasons, and travel restrictions.

### Recommendation for Training of Trainers

The trainer and counselors emphasized the need for “encouragement and feedback … to support each other in peer groups (S)… and more role-play and practice” (C), to be able to train others. Ongoing supervision was considered important, as it challenged counselors to “move beyond their comfort zone (T).” The supervisor was specific in recommending ‘training of trainers’ sessions to bring together counselors, “four from Nablus, East Jerusalem/Ramallah, Jenin and Hebron to present this training as a rehearsal to empower them to work on what they face in the field.”

In summary, as counselors made most statements, this appropriately gave weight within the analysis to counselor experience. Counselor themes showed an inter-relatedness of the functions of supervision, however, formative and normative functions were spoken of most. The restorative function was needed immediately after training and subsequently led to reduced stress and increased confidence in program delivery. This is perhaps not surprising given counselors were new to the program and had a short period of time before delivering TRT. The supervisor tended to rate gains for counselors only slightly lower than counselors rated themselves. While there was high commonality of themes between counselor and supervisor, the supervisor reported on a range of unique themes, indicating a wider perspective. Trainer themes, covered the inter-dependence of the three functions in PGS, in order for skills to transfer from training to program delivery and concerns for barriers that could get in the way. PGS was seen as a way of addressing some of these barriers.

## Discussion

This case study, of the unique development of PGS to TRT for counselors in villages in Palestine, contrasted counselor, supervisor and trainer perspectives. Reports by all three participant groups indicate that even in a context of military violence, PGS provides a reflective process inclusive of formative, normative and restorative functions. Although many of the benefits of PGS reported by counselors and supervisor can be found in supervision literature (increasing clarity of practice, sharing learnings, growth in confidence, program protocol adherence, reduced stress, and improved listening and group communication skills; Hawkins and Shohet 2006), comments indicated counselors and supervisor held unique perspectives on PGS within a context of occupation. For example, despite years of delivering service, this was counselors’ first experience of supervision. Not surprisingly, counselors reported anxiety in the early stages, however, it is apparent from counselor and supervisor reports that counselors became increasingly comfortable with PGS. A particular benefit to counselors in Palestine was having supervision available locally. This appears to have avoided many of the problems of travel, such as the need for permits and the risk of checkpoints suddenly closing, resulting in the cancelation of meetings. Likewise, as salaries are regularly unpaid (Barron and Abdallah 2014), local supervision avoided the added pressure of travel costs. Within a geographically fragmented context, then, PGS with counselors from nearby schools appears to have reduced counselor isolation, fulfilling a restorative function.

Counselors’ reported an increased capacity to listen to each other and respect each other’s work. Specifically to Palestine, PGS provided a formative context for counselors to discuss and practice the skills of group work in moving from the narrow practice of form filling to one of interactive group activities. Of particular relevance, PGS facilitated counselors’ self-disclosure within a context of distrust. This is an important restorative and normative issue, as the use of informants in the region has embedded suspicion into community and professional relationships (Barron and Abdallah 2014). PGS then, appears to have created emotionally safe spaces in a context of adversity for counselors to share their work-based thoughts and feelings. Likewise, PGS was reported as enabling self-disclosure for women. This is challenging of cultural gender norms, as women living in a male dominated Palestinian culture are not expected to share personal information in a mixed sex public sphere. Indeed, in some parts of Palestinian society, it would viewed as a stigma for unmarried woman to have contact with a man beyond the family (Hasso 2005).

The supervisor raised a number of unique perspectives on PGS. Counselors, for example, were reported as using prayer in their preparation of PGS and program delivery. In contrast to the West, spiritual practices of this kind, and their impact on effectiveness, are rarely discussed. The field of spiritual counseling, however, would be an exception (Stebnicki 2006). The supervisor also highlighted, that despite the demoralizing context of war, PGS provided a process that facilitated counselor motivation through increased sense of purpose and support. Professional and cultural isolation reduced because of counselor contact with national and international organizations; that is, PGS provided a context for enwalled counselors to have productive communication with professionals beyond Palestine. Finally, the trainer perceived PGS as a useful follow-up to address emotive issues that had arisen in training, incorporating both formative and restorative functions. It would appear that contributions from different perspectives identified a wide range of benefits that PGS can bring to a context of violent military occupation.

Similarly to the benefits of PGS, many barriers reported by counselors are identified in current literature. This included experiencing uneasiness as a result of reflecting on the limitations of past practice, discomfort during the process of learning new knowledge and skills, and counselor cautiousness in being sufficiently skilled to deliver a program for the first time (Proctor 2004). Some of the reported barriers of PGS were, however, unique to the context of military occupation. These barriers were numerous including unpaid salaries resulting in school strikes and closures; no funds for other formative or restorative activities; military incursions stopping or interrupting sessions; and spontaneous unpredictable road blocks requiring plans for meetings to be changed. Despite these barriers, PGS appears to have been sufficiently valued by counselors to sustain participation.

In contrast, there was a significant difference between trainer, counselors and supervisor regarding counselor capacity to train other counselors. The trainer reported counselors overestimated their need for further input, given their extensive experience of working with traumatized adolescents. Despite PGS, however, only 50% of counselors felt ready to train others. Counselors and supervisor identified a range of factors in Palestine underpinning this lack of self-efficacy: the lack of sufficient training in psychology and social work; the geographical, social and political isolation and division felt as professionals and as individuals; the cumulative violence, trauma and daily humiliations experienced by themselves, relatives and the adolescents and families; the unpredictability of daily life; and the nature and extent of complex trauma in adolescents. In summary, counselors and supervisor reported that the daily events of military occupation left counselors’ feeling professionally and personally demoralized to such an extent that there was little confidence in going beyond program delivery to train peers. The lack of receptiveness of some non-trained counselors was reported as low because of similar issues.

### Limitations

Case studies are criticized for the singularity of their focus and the limited generalizability to other situations. On the other hand, they provide a richness of information about participant experience and their meanings in particular contexts (Orne and Bell 2015). In this study, unique accounts were recorded from counselors, supervisor and trainer. Given there were seven counselors, this may not have been sufficient for saturation of issues to emerge, again limiting generalization of findings. Orne and Bell (2015) suggest sample sizes of 12–14 are needed for no new significant issues to emerge. It is unknown if non-participants held significantly different views. The case study was conducted in rural villages; the experience may be different for school counselors in cities, where there may be more resources. With researchers conducting supervision training, this may have led to reluctance on the part of counselors to share their negative PGS experiences. As one of the researchers was the supervisor, counselor response may have included appreciation for supervision received, and less critical of skilled PGS. Further, as the supervisor was interviewed as a participant, he may have been more positive about his experiences. To address this concern, much of the supervisor’s discourse has been reported to enable reader analysis of contribution. Interviews were conducted post-supervision only. Pre-supervision interviews would have enabled an evaluation of change in counselor perspective before and after PGS. Although there were rating scales and frequency counts of codes, the study did not seek to quantify the nature of counselor change. Quasi-qualitative analysis can be criticized for being overly compartmentalized and frequency counts may not necessarily convey a sense of greater importance of meaning.

### Conclusion

The case study found that, despite the small number of supervision sessions and the ongoing stressors of military occupation, PGS provided a reflective process inclusive of formative, normative and restorative functions. Benefits did not simply fit into one function or another. Rather the interaction of all three functions seems to have been significant. Benefits and barriers of PGS mirrored both issues within PGS literature, and unique issues within a context of violent military occupation. Locally available PGS was reported as essential within a geographically fragmented context. Counselor self-disclosure within PGS led to a trusting environment in contrast to military practices and gender norms. The use of prayer in preparation of supervision was an unexpected finding that needs more research. Finally, indications are there are too many situational war stressors undermining counselor confidence for PGS to enable counselors’ to train their peers in TRT.

### Recommendations for Practice and Policy

PGS should continue to be available locally for counselors delivering TRT in the West Bank. This should be augmented by skilled supervisor sessions. Opportunities for individual as well as group supervision should be explored. Responding to counselor and supervisor requests, a trainer of trainers should be developed to enable counselors to train colleagues across the West Bank. PGS should be expanded to be trialed for counselors in city schools. Finally, PGS cannot compensate for addressing the political and military barriers identified by counselors. Peaceful political solutions need to be sought.

PGS training and supervised practice needs to be embedded within Palestinian Authority Ministry of Education policy. Schools need guidelines on recognizing the value of PGS including releasing counselors for supervision. Guidelines should also facilitate ongoing evaluation of PGS and their practice. This would enable a longitudinal perspective on impact. Funding, for example, from international NGOs, needs explored to sustain this.

### Recommendations for Research

Interviews could be held before and after PGS as a next step in evaluating effectiveness. Beyond case studies, quasi-experimental and experimental methodologies are needed to quantify the outcomes of PGS for counselors and subsequently for adolescents who experience TRT. Larger sample populations need to be in city and rural locations. Finally, the impact of prayer on PGS effectiveness needs explored.
